# Prenatal Stress Enhances Excitatory Synaptic Transmission and Impairs Long-Term Potentiation in the Frontal Cortex of Adult Offspring Rats

**DOI:** 10.1371/journal.pone.0119407

**Published:** 2015-03-06

**Authors:** Joanna Sowa, Bartosz Bobula, Katarzyna Glombik, Joanna Slusarczyk, Agnieszka Basta-Kaim, Grzegorz Hess

**Affiliations:** 1 Department of Physiology, Institute of Pharmacology, Polish Academy of Sciences, Krakow, Poland; 2 Department of Experimental Neuroendocrinology, Institute of Pharmacology, Polish Academy of Sciences, Krakow, Poland; 3 Institute of Zoology, Jagiellonian University, Krakow, Poland; Radboud University, NETHERLANDS

## Abstract

The effects of prenatal stress procedure were investigated in 3 months old male rats. Prenatally stressed rats showed depressive-like behavior in the forced swim test, including increased immobility, decreased mobility and decreased climbing. In *ex vivo* frontal cortex slices originating from prenatally stressed animals, the amplitude of extracellular field potentials (FPs) recorded in cortical layer II/III was larger, and the mean amplitude ratio of pharmacologically-isolated NMDA to the AMPA/kainate component of the field potential—smaller than in control preparations. Prenatal stress also resulted in a reduced magnitude of long-term potentiation (LTP). These effects were accompanied by an increase in the mean frequency, but not the mean amplitude, of spontaneous excitatory postsynaptic currents (sEPSCs) in layer II/III pyramidal neurons. These data demonstrate that stress during pregnancy may lead not only to behavioral disturbances, but also impairs the glutamatergic transmission and long-term synaptic plasticity in the frontal cortex of the adult offspring.

## Introduction

An increasing body of recent evidence indicates that early life adverse experiences can affect brain development and may be involved in the pathogenesis of psychiatric disorders including depression (reviewed in [1,2,3]). Prenatal stress in rats represents one of the established animal models of depression. In the adult offspring of females subjected to stress during pregnancy, increased immobility time in the forced swim test, sleep and cognitive functions disturbances, decreased sexual behavior and abnormalities in the functioning of the immune and neuroendocrine systems have been observed [4,5,6,7,8]. It has been shown that prenatal stress results in a decrease in the amount of NMDA receptor subunits and a reduced potential for long-term potentiation (LTP) in the hippocampal CA1 area of mice [9] and rats [10,11,12]. It has been postulated that these effects may underlie deficits in spatial learning and memory, evident in prenatally stressed animals [9,10,11]. Moreover, some data show that spatial learning impairment may be partly related to the disturbances in the adult neurogenesis in the dentate gyrus (DG) of prenatally stressed rats [13]. On the other hand, mild prenatal stress may facilitate neurogenesis and LTP in the DG of adult rats [14].

While it has been shown that the prenatal stress procedure may disturb brain development and the organization of neuronal circuits in the prefrontal cortex [15], (reviewed in [16]), the impact of prenatal stress on the function of other cerebral cortical areas has not been investigated yet. In the rat brain, the primary motor cortex (M1), identified through the occurrence of motor responses to low intensity intracortical microstimulation, corresponds to the lateral agranular area of the frontal cortex [17]. It has been established that in adult rat, the M1 expresses a potential for plastic reorganizations (reviewed in [18]). Interestingly, the M1 reorganization, involving the expansion of motor representations, has been shown to accompany motor skill learning [19,20]. Reorganization of the M1 may engage LTP-like mechanisms [21,22,23].

Based on these studies, it is plausible that prenatal stress may affect the glutamatergic synaptic transmission and plasticity of the primary motor cortex. To test this hypothesis, we sought to determine the effects of prenatal stress procedure on field potentials (FPs) and spontaneous excitatory postsynaptic currents (sEPSCs), as well as on the LTP, in the frontal cortical area M1 of the adult offspring rats.

## Materials and Methods

### Animals

Adult Sprague—Dawley rats were housed under controlled light/dark cycle (light on: 7:00–19:00 h), with free access to tap water and standard food (Special Diet Services, UK). Two weeks after arrival, vaginal smears were taken daily from the female rats to determine the phase of the estrous cycle. On the proestrus day, females where placed with males for 12 h and afterwards the presence of sperm in vaginal smears was checked. Pregnant rats were randomly assigned to the control and experimental group.

### Ethics Statement

The experimental procedures were approved by the Animal Care and Use Committee at the Institute of Pharmacology, Polish Academy of Sciences, and were carried out in accordance with the European Community guidelines for the use of experimental animals and the national law. All efforts were made to minimize animal suffering and the number of animals used.

### Prenatal stress procedure

Pregnant female rats were subjected daily to three stress sessions from the 14th day of pregnancy until the delivery, as described previously [6,8]. At 09.00, 12.00 and 17.00 h rats were placed in plastic cylinders (diameter: 7 cm, length: 12 cm) and exposed to a bright light (150 W) for 45 min. Control pregnant females were left undisturbed in their home cages. Only male offspring (21 day-old) from litters containing 8–10 pups with a similar number of males and females were used for the experiments. Rats were housed in groups of five animals per cage (1–2 animals from each litter) under standard conditions. At three months of age, the offspring of stressed and unstressed (control) mothers were first tested for behavioral changes, and later used for electrophysiological recordings of FPs. A separate group of 10 animals (5 prenatally stressed and 5 control) was used for whole-cell recordings.

### Forced swim test (FST)

The control and prenatally stressed rats (n = 10 in each group) were individually subjected to two trials during which they were forced to swim in a cylinder (40 cm high, 18 cm in diameter) filled with water (25°C) up to a height of 35 cm. There was a 24-h interval between the first and the second trial. The first trial lasted for 15 min, while the second trial was carried out for 5 min. The total duration of immobility, mobility (swimming) and climbing was measured by the observer throughout the second trial [24].

### Brain slice preparation

Rats were anesthetized with isoflurane (Aerrane, Baxter) and decapitated. Their brains were immersed in an ice-cold artificial cerebrospinal fluid (ACSF) containing (in mM): 130 NaCl, 5 KCl, 2.5 CaCl_2_, 1.3 MgSO_4_, 1.25 KH_2_PO_4_, 26 NaHCO_3_ and 10 D-glucose, bubbled with the mixture of 95% O_2_ and 5% CO_2_. Frontal cortical slices (thickness: 420 μm) were cut from one of the hemispheres using a vibrating microtome (Leica VT1000S).

### Whole-cell recording

Individual slices were placed in the recording chamber mounted on the stage of the Axioskop (Zeiss) microscope and superfused at 2 ml/min with modified ACSF of the following composition (in mM): 132 NaCl, 2 KCl, 1.25 KH_2_PO_4_, 26 NaHCO_3_, 1.3 MgSO_4_, 2.5 CaCl_2_, and 10 D-glucose 10, bubbled with 95%O_2_-5%CO_2_ (temperature: 32 ± 0.5°C). Recording pipettes were pulled from borosilicate glass capillaries (Harvard Apparatus) using Sutter Instrument P97 puller. The pipette solution contained (in mM): 130 K-gluconate, 5 NaCl, 0.3 CaCl_2_, 2 MgCl_2_, 10 HEPES, 5 Na_2_-ATP, 0.4 Na-GTP, and 1 EGTA (osmolarity: 290 mOsm, pH = 7.2). Pipettes had open tip resistance of approx. 6 MΩ. Pyramidal cells were sampled from the sites located approx. 2.5 mm lateral to the midline and approx. 0.3 mm below the pial surface as described previously [25]. Signals were recorded using the SEC 05LX amplifier (NPI), filtered at 2 kHz and digitized at 20 kHz using Digidata 1440A interface and Clampex 10 software (Molecular Devices).

### Field potential recording and LTP induction

The slices were placed in the recording chamber of an interface type and superfused at 2.5 ml/min with warm (32 ± 0.5°C), modified ACSF (see above). A concentric bipolar stimulating electrode (FHC, USA) was placed in cortical layer V. Stimuli of 0.033 Hz frequency and duration of 0.2 ms were applied using a constant-current stimulus isolation unit (WPI). Glass micropipettes filled with ACSF (2–5 MΩ) were used to record field potentials. Recording microelectrodes were placed in cortical layer II/III. The responses were amplified (EXT 10-2F amplifer, NPI), filtered (1 Hz-1 kHz), A/D converted (10 kHz sampling rate) and stored on PC using the Micro1401 interface and Signal 2 software (CED).

A stimulus-response (input-output) curve was made for each slice. To obtain the curve, stimulation intensity was gradually increased stepwise (15 steps; 5–100 μA). One response was recorded at each stimulation intensity. In the first set of slices, the recording was initially performed in standard ACSF followed by 25 min perfusion with modified ACSF containing 5 μM (±) -2-amino-4-methyl-5-phosphono-3-pentenoic acid (CGP-37849, Tocris), to block NMDA receptors. Subsequently, to unblock NMDA receptors and to block AMPA/kainate receptors, the slice was perfused for 25 min with ACSF devoid of Mg^2+^ ions and containing 10 μM 2,3-dioxo-6-nitro-1,2,3,4-tetrahydrobenzo[f]-quinoxaline-7-sulfonoamide (NBQX disodium salt, Tocris) [26]. In the second set of slices, incubated only in standard ACSF, stimulation intensity was adjusted to evoke a response of 30% of the maximum amplitude. LTP was induced by theta burst stimulation (TBS). TBS consisted of ten trains of stimuli at 5 Hz, repeated 5 times every 15 s. Each train was composed of five pulses at 100 Hz. During TBS pulse duration was increased to 0.3 ms.

### Data analysis

Statistical analysis of behavioral data was carried out using one-way ANOVA followed by the Duncan's post hoc test, when appropriate.

The stimulus-response curves for each slice were fit with the Boltzmann equation: V_i_ = V_max_/(1+exp ((u-u_h_)/-S)). The following parameters were compared: the maximum field potential amplitude (V_max_), the stimulation intensity evoking a field potential of half-maximum amplitude (u_h_) and the factor proportional to the slope of curve (S). The threshold stimulation was determined as the stimulus intensity necessary to evoke a field potential of approximately 0.1 mV in amplitude. First 15 min of the recording was the baseline (100%), against which subsequent measurements were normalized. As LTP in this preparation develops gradually [27] the amount of LTP was determined as an average increase in the amplitude of FPs, after stabilization of responses (between 60–75 min after TBS) relative to baseline. The results are expressed as the means ± SEM. Statistical analysis of electrophysiological data was carried out using the Student's t–test or the Mann-Whitney U test.

## Results

### Effects of prenatal stress on the forced swimming

**Fig 1:**
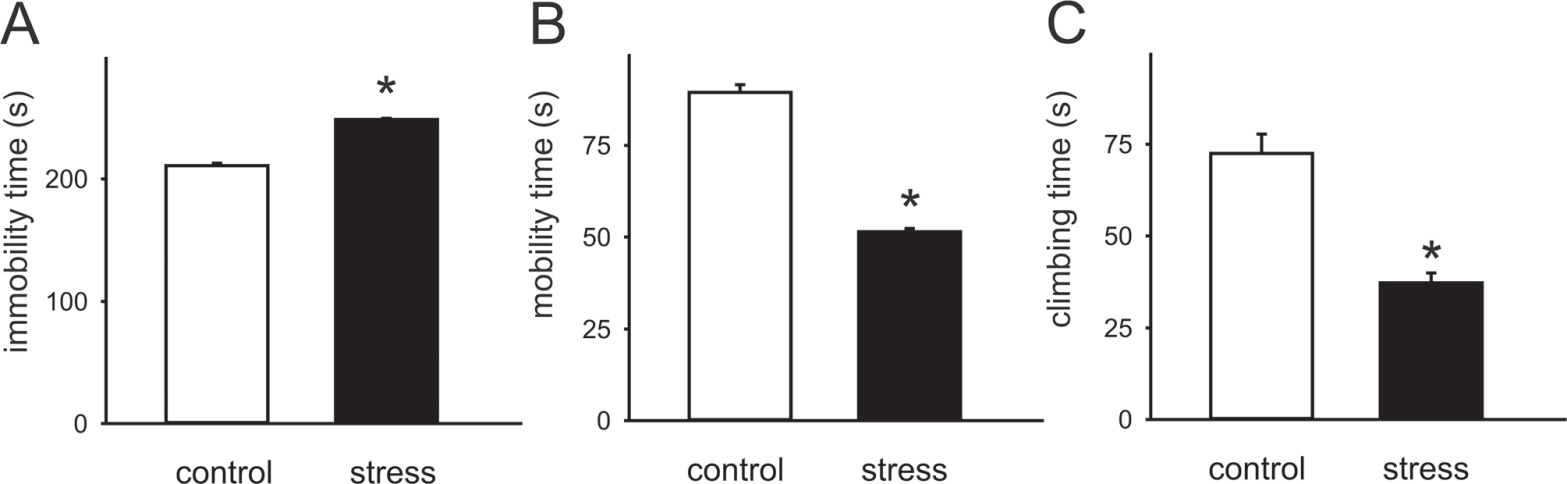
The effects of prenatal stress on immobility (A), mobility (B) and climbing (C) in the forced swim test. Shown are mean values ± SEM. * p < 0.05. White bars represent control rats (n = 10) and black bars—prenatally stressed rats (n = 10).

Prenatally stressed rats demonstrated significantly prolonged immobility time (F(1,18) = 288.33; p < 0.05; Fig. 1A) during the second trial of the forced swim test, consistent with what has been reported previously [6,8]. Moreover, compared with control animals, prenatally stressed rats exhibited shortened swimming time (F(1,18) = 279.82; p < 0.05) and climbing time (F(1,18) = 35.35; p < 0.05; Fig. 1B, C).

### Effects of prenatal stress on field potentials

**Fig 2:**
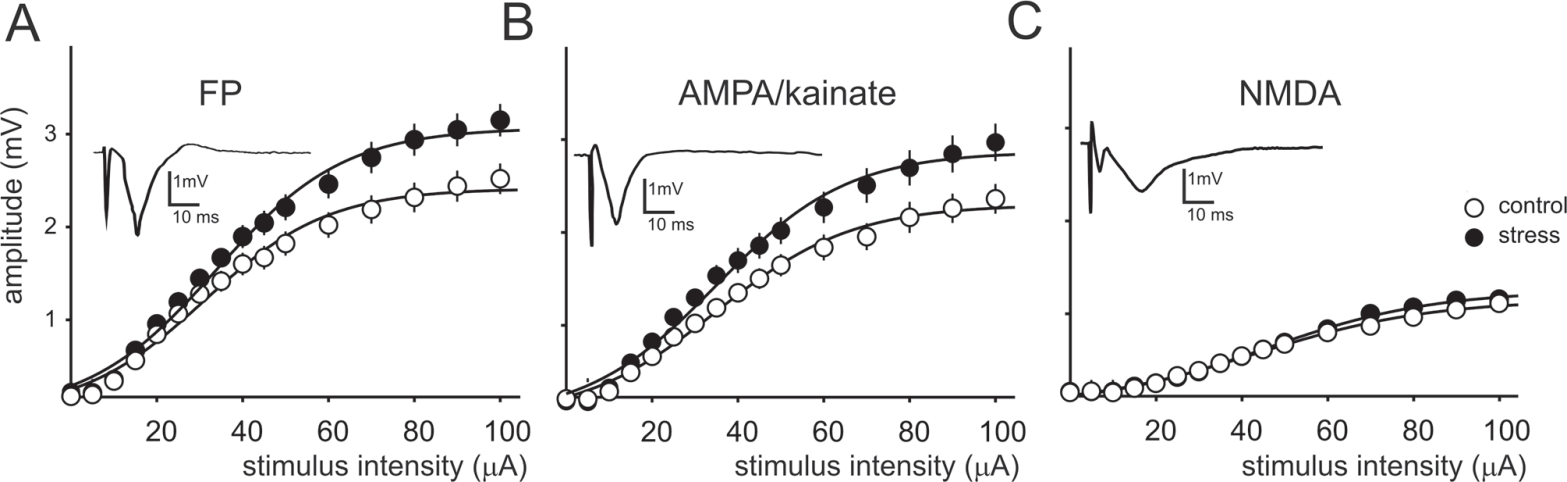
Prenatal stress increases the amplitude of the AMPA/kainate component of field potentials (FPs). Graphs illustrate the effect of prenatal stress on the relationship between stimulus intensity and the mean response amplitude (± SEM) on (A) composite FPs (in normal ACSF), (B) the AMPA/kainate component and (C) the NMDA component of FPs. Black circles—slices (n = 8 to 12) obtained from stressed rats (n = 10), white circles—control slices (n = 8 to 12, obtained from 10 rats). Lines represent fits to the Boltzmann equation (see: Table 1). Insets: examples of representative responses.

Analyses of FPs evoked in the standard ACSF in slices obtained from prenatally stressed rats, revealed a shift of the relationship between stimulus intensity and FP amplitude (input-output curve) compared with the slices obtained from control rats (Fig. 2A). In slices originating from stressed rats also the AMPA/kainate component of FPs, recorded after addition of 5 μM CGP-37849 to the ACSF, showed an increase in the input-output relationship (Fig. 2B). In contrast, responses recorded in the ACSF devoid of Mg^2+^ ions and containing 10 μM NBQX to isolate the NMDA receptor-mediated component of FPs, did not differ from control (Fig. 2C). Thus, in prenatally stressed rats, the mean amplitude ratio of the NMDA to the AMPA/kainate component (evoked by stimulation in the range: 50–100 μA) was significantly decreased compared to control rats (0.43 *±* 0.02 vs. 0.52 *±* 0.01, respectively, p = 0.006). Parameters characterizing input-output curves of FPs as well as AMPA/kainate and NMDA components, calculated using the Boltzmann fits, are summarized in Table 1.

**Table 1:** Effects of prenatal stress on the parameters characterizing stimulus-response curves of recorded responses calculated using the Boltzmann fits.

		Threshold (μA)	V_max_ (mV)	u_h_ (μA)	S	n
**composite field potentials**	**control**	13.26 ± 0.97	2.45 ± 0.16	33.38 ± 1.5	13.97 ± 0.8	24
	**prenatal stress**	11.73 ± 0.80	3.08 ± 0.17**	34.29 ± 1.31	14.15 ± 0.6	24
**AMPA/kainate component**	**control**	14.13 ± 1.11	2.31 ± 0.16	36.63 ± 1.17	15.44 ± 0.83	24
	**prenatal stress**	13.95 ± 2.05	2.88 ± 0.19**	35.76 ± 1.4	15.04 ± 0.56	20
**NMDA component**	**control**	22.35 ± 2.42	1.38 ± 0.11	48.23 ± 3.11	16.85 ± 2.41	16
	**prenatal stress**	22.00 ± 2.00	1.55 ± 0.1	50.00 ± 3.1	14.78 ± 1.3	13

Data are presented as means ± SEM. Threshold, stimulus intensity evoking responses of approx. 0.1 mV in amplitude; V_max_, maximum amplitude; u_h_, half maximum stimulation, S, factor proportional to the slope of the curve; n, number of slices.

** p < 0.01.

### Effects of prenatal stress on pyramidal cell membrane excitability and postsynaptic currents

**Fig 3:**
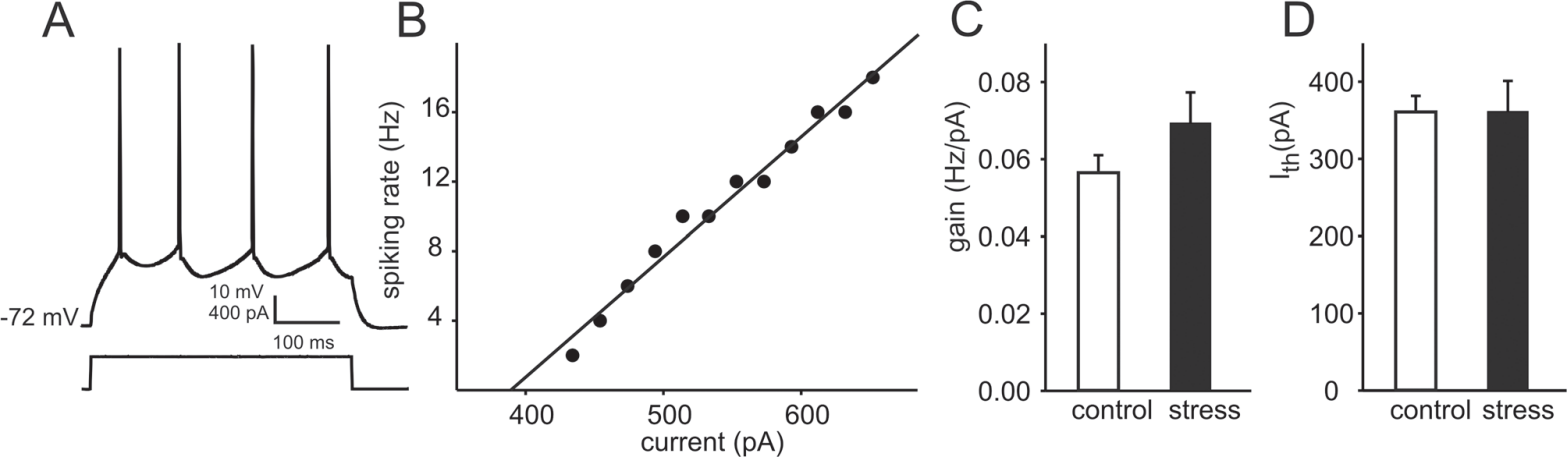
Prenatal stress does not modify the intrinsic excitability of layer II/III pyramidal neurons. (A) Example of a response of a cell from a stressed rat (upper trace) to a depolarizing current pulse (lower trace). (B) Example of an injected current vs. spiking rate relationship in a cell shown in (A). (C) The mean firing threshold (± SEM) and (D) the mean gain (a slope of injected current vs. spiking rate relationship; ± SEM) of pyramidal neurons prepared from control (white bars; 19 cells from 5 rats) and prenatally stressed animals (black bars; 16 cells from 5 rats). The differences are not significant.

Whole-cell recordings were obtained from layer II/III neurons exhibiting a regular spiking firing pattern in response to a depolarizing current pulse (Fig. 3A) and no spontaneous spiking activity at the resting membrane potential. There were no significant differences between neurons originating from prenatally stressed and control animals either in the resting membrane potential (−74.9 ± 0.7 vs. −75.3 ±0.6 mV, respectively, p = 0.684) or the input resistance (44.9 ± 3.2 vs. 42.3 ± 3.1 MΩ, respectively, p = 0.563). Analyses of the relationship between injected current and firing rate (Fig. 3B) demonstrated that prenatal stress did not modify the intrinsic excitability of frontal cortical pyramidal neurons (Fig. 3C, D).

**Fig 4:**
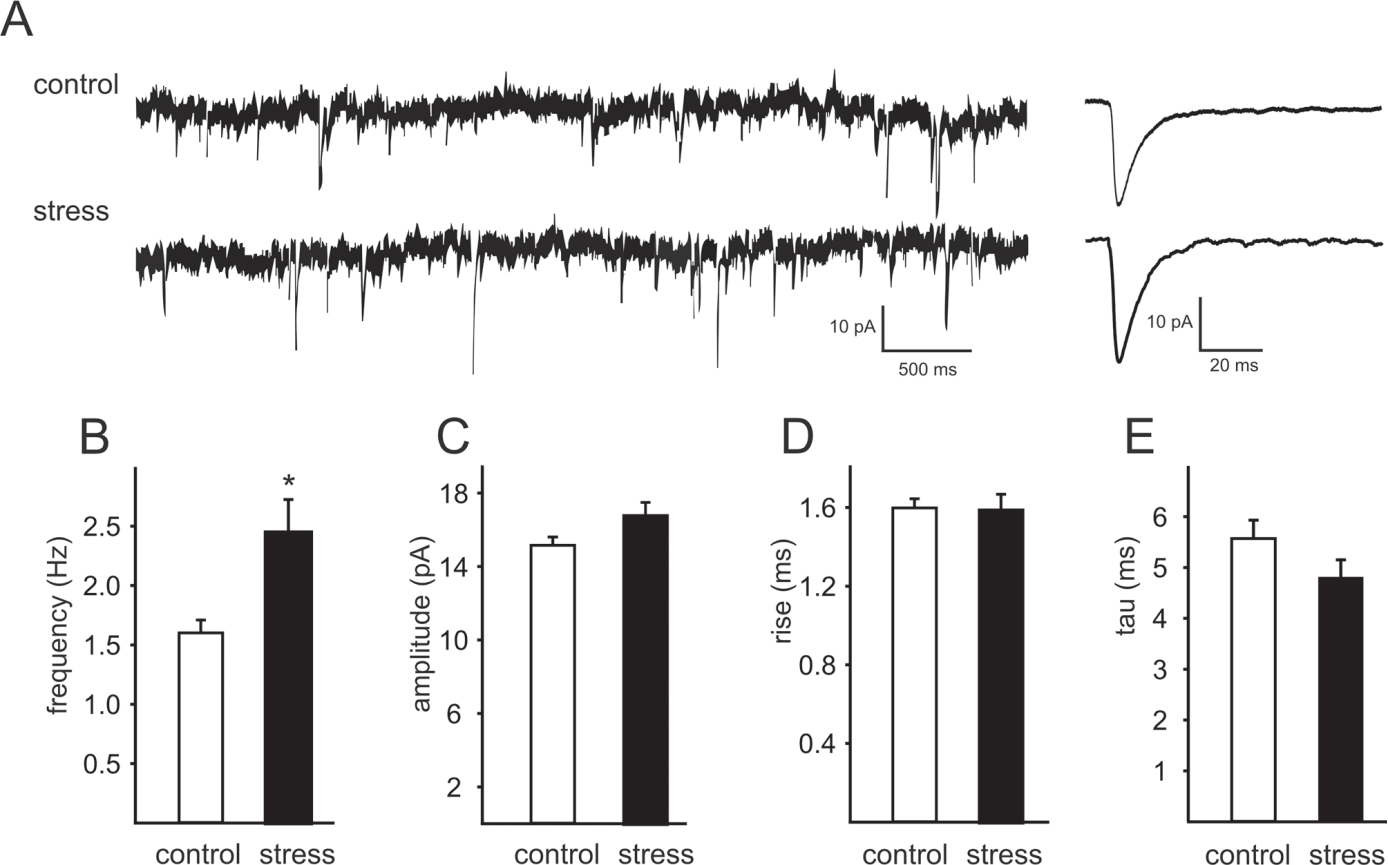
Prenatal stress increases the frequency of sEPSCs recorded from layer II/III pyramidal neurons. (A) Typical examples of raw records from a control neuron (upper trace on the left) and a neuron originating from prenatally stressed rat (lower trace on the left). Traces to the right represent averages of all individual sEPSCs detected during 4 min recordings from a control neuron (upper trace) and a neuron originating from a stressed rat (lower trace). Bar graphs illustrate the effect of prenatal stress on (B) the mean frequency, (C) the mean amplitude, (D) the rise time and (E) the decay time constant of sEPSCs. In B-E, the error bars represent SEM; * p < 0.05. White bars represent neurons (n = 19) originating from control rats (n = 5) and black bars—neurons (n = 16) from prenatally stressed animals (n = 5).

Spontaneous EPSCs were recorded at the holding potential of −76 mV as inward currents (Fig. 4A). In the cells originating from prenatally stressed rats the mean frequency of sEPSCs was significantly higher in comparison to that in the cells from control animals (2.44 ± 0.28 vs. 1.60 ± 0.11 Hz, p = 0.022; Fig. 4B). The stress did not affect the mean amplitude, the mean rise time and the mean decay time constant of sEPSCs (Fig. 4C-E).

### Effects of prenatal stress on long-term potentiation

**Fig 5:**
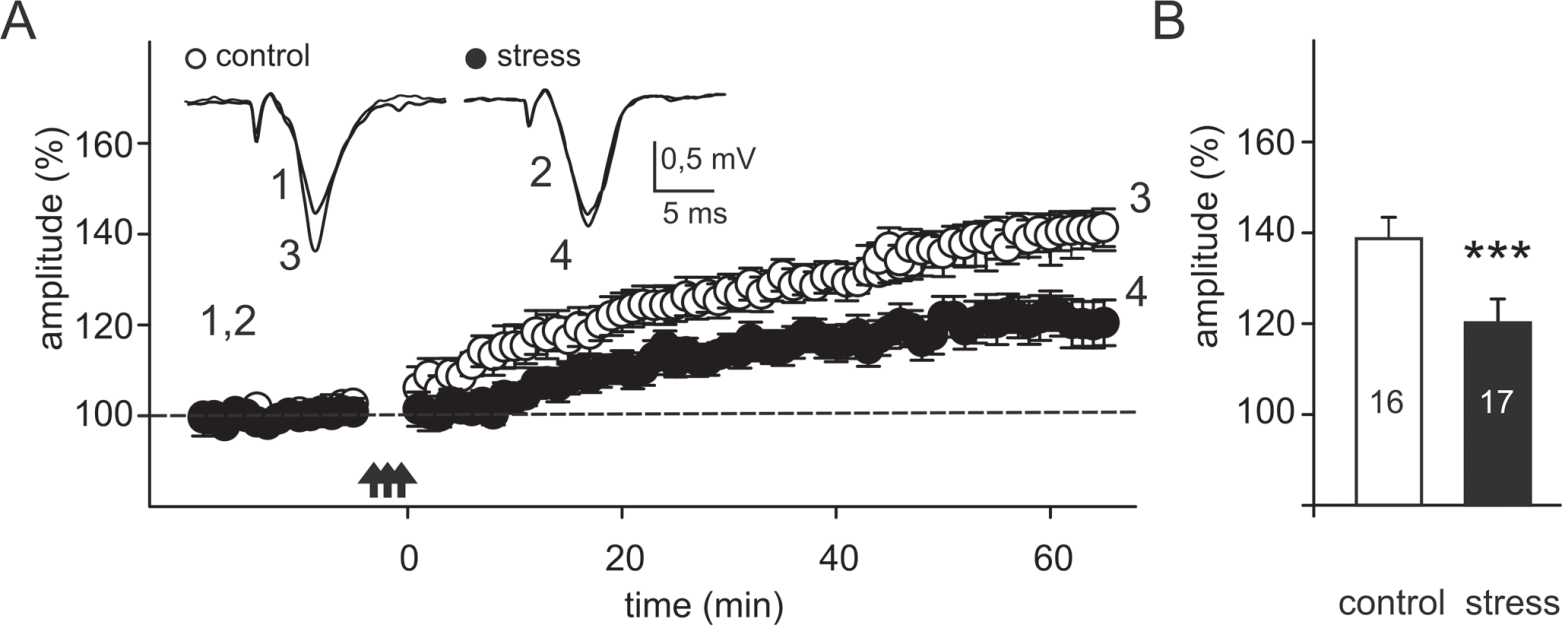
Prenatal stress impairs the induction of LTP. (A) Plot of the amplitude of FPs (mean ± SEM) recorded in slices obtained from control rats (white circles) and from rats subjected to prenatal stress (black circles). Arrows denote the time of theta-burst stimulation (TBS, repeated 3 times). Insets show superposition of FPs recorded during representative experiments before and after TBS at times indicated by numbers. (B) Mean (± SEM) amplitude of FPs recorded between 60–75 min after TBS in slices prepared from control (n = 10) and prenatally stressed (n = 10) rats. The numbers on the bars indicate the numbers of slices in each group. *** p < 0.001; Mann-Whitney U test.

In the slices prepared from control rats the mean amplitude of FPs, measured between 60–75 min after TBS, was 139.9 ± 3.9% of baseline (Fig. 5A). LTP was significantly attenuated in the slices obtained from stressed animals (121.0 ± 4.8%; p < 0.001; Fig. 5B).

## Discussion

The results of the present study demonstrate that prenatal stress induces an enhancement of the excitatory input to layer II/III pyramidal cells and an impairment of long-term synaptic plasticity in the lateral agranular area of the frontal cortex (M1), evident in adult male offspring of stressed pregnant female rats. We have also confirmed that in the offspring of rat dams that were stressed during the last week of pregnancy, behavioral disturbances are present [6,8]. Prenatally stressed rats exhibit not only an increase in immobility time, but also a decrease in swimming and climbing behavior in the modified Porsolt test. Mobility and immobility scores in this test appear to be dependent primarily on activity of the medial prefrontal cortex [28]. However, there is an evidence that exposure of rats to forced swim test results in activation of larger areas of the neocortex, including prefrontal, orbital, cingulate and motor cortex [29]. Undamaged cortical representations of forelimbs are necessary to maintain the normal swim pattern in rats [30]. Thus, it is conceivable that the observed prenatal stress-related behavioral disturbances may result, in part, from functional alterations within the motor cortex.

The contribution of NMDA and AMPA/kainate receptor-mediated components of FPs to cortical excitatory synaptic transmission was assessed, using the specific antagonists of these two glutamate receptor groups [31]. Compared to control preparations, in slices obtained from prenatally stressed rats the AMPA/kainate component was enlarged to a similar degree as the composite FPs, consistent with the fact that the contribution of NMDA receptors to synaptic transmission in baseline conditions, in the ACSF containing 1,3 mM Mg^2+^, is small [32]. A lack of prenatal stress-induced change in the magnitude of the pharmacologically-isolated NMDA component of FPs, recorded in the absence of Mg^2+^ ions, resulted in an overall decrease in the ratio of the NMDA to the AMPA/kainate component. A relatively smaller contribution of NMDA receptors to synaptic responses, evoked by TBS of a given intensity, might be one of the reasons of an impairment of LTP, evident after prenatal stress, as LTP in the M1 is NMDA receptor-dependent [33].

Present data demonstrate that prenatal stress resulted in an increased frequency of spontaneous EPSCs recorded from layer II/III pyramidal neurons. No modification of the membrane excitability of these cells was evident. In our previous study we demonstrated a lack of significant change in the mean frequency of sEPSCs after the blockade of Na^+^ channels by tetrodotoxin in slices of the frontal cortex [34]. Thus, a majority of recorded sEPSCs correspond to miniature EPSCs (mEPSCs), whose frequency is unrelated to the spiking activity of the presynaptic cell (see also: [35]). Changes in the frequencies of sEPSCs and mEPSCs are regarded as indicative of changes in the probability of neurotransmitter release and/or changes in the number of neurotransmitter release sites (e.g. [36]). Thus, our present data suggest that prenatal stress results in an enhanced glutamate release from presynaptic terminals. It should be noted that recordings of postsynaptic currents were conducted in the presence of 1.3 mM Mg^2+^ at the holding potential of -76 mV and under these conditions the contribution of NMDA receptor-mediated currents is unlikely. Therefore, increased frequency of sEPSCs, reflecting enhanced glutamate release, is consistent with the observed increase in the amplitude of the AMPA/kainate receptor-mediated component of FPs. An apparent lack of change in the amplitude of the NMDA component of FPs, accompanying increased neurotransmitter release, may be explained by a stress-related reduction in the reactivity of postsynaptic NMDA receptors, while the reactivity of AMPA/kainate receptors remained unchanged. This effect would explain the observed decrease in the ratio of the NMDA to the AMPA/kainate components. In fact, a decrease in the expression level of NMDA receptor subunits has previously been documented in the brain of prenatally stressed rats [37]. A lack of change in the mean amplitude of sEPSCs in cells originating from prenatally stressed animals indicates that the reactivity of postsynaptic AMPA/kainate receptors was not modified by stress. Notably, no change in the expression level of the phosphorylated GluA1 subunit of the AMPA receptor was observed in the frontal cortex of prenatally stressed male rats [38].

Altogether, these data suggest that prenatal stress modifies baseline glutamatergic transmission in the motor cortex both at the presynaptic (enhanced glutamate release) and postsynaptic (decreased NMDA receptor reactivity) levels. It has been shown that in neocortical preparations, synaptic connections with greater initial strengths are less likely to undergo plasticity, whereas weaker connections tend to potentiate by presynaptic mechanisms (e.g. [39,40]). It is conceivable that prenatal stress may activate presynaptic mechanisms which normally support the expression of LTP, leading to a lowered potential for activity-dependent synaptic potentiation.

There is some evidence that prenatally stressed rats display enhanced and prolonged hypothalamic-pituitary-adrenal (HPA) axis responses to physical and psychological stressors in adulthood (reviewed in [2]). Furthermore, abnormalities in the activity of the HPA axis appear to be permanent because we observed that prenatally stressed adult rats have elevated corticosterone levels and impaired glucocorticoid receptor (GR) function in the brain, resulting from a decreased concentration of immunophilin FKBP51 [8]. In our other study, in the frontal cortex of young adult rats subjected to repeated restraint stress, an increase in the amplitude of glutamate-mediated extracellular field potentials and an impairment of LTP have been demonstrated [41]. Similar results have been obtained after repeated corticosterone administration [27]. Therefore, it is likely that the effects, observed in the course of the present study, may result from a higher corticosterone secretion in prenatally stressed animals in response to stress and/or disturbed circadian rhythm of corticosterone secretion [8,42,43].

On the other hand, the role of neurotrophic factors in glutamatergic transmission and long-term synaptic plasticity disturbances in the frontal cortex should be considered. Recently, insulin-like growth factor (IGF-1) has gained great attention in this respect. The uniqueness of IGF-1 manifests itself in the ability to antagonize the neuroinflammatory response [44]. That way IGF-1 may also modulate the HPA axis activity through the influence on cytokine release. Our recent research has demonstrated a decrease in the IGF-1 level, IGF-1 receptor phosphorylation as well as the dysregulation of the IGF-1 binding protein network in the frontal cortex of adult, prenatally stressed rats [4,5]. By acting as a neurotrophic factor, IGF-1 stimulates the growth and differentiation of sensory, motor and sympathetic neurons and is the only growth factor that enhances the regeneration of both sensory and motor nerves in adult animals [45]. Importantly, there is some evidence that IGF-1 may directly influence glutamatergic synaptic transmission, as it has been demonstrated, among other things, that acute IGF-1 administration enhances AMPA and NMDA receptor-mediated field potentials in the slices of the rat hippocampus [46]. Since IGF-1 and some other proteins of the insulin-like growth factor family modulate synaptic transmission and plasticity at different sites via various direct or indirect pathways (reviewed in: [47]), it is conceivable that the diminished level of IGF-1 in the frontal cortex of prenatally stressed rats may be responsible, in part, for the observed alterations.

Data indicate that IGF-1 exerts its biological functions in the brain through the IGF-1 receptor, which mediates phosphorylation of the insulin receptor substrate (IRS) proteins [4,48]. IRS-1 and IRS-2 are most highly expressed regulatory proteins in the rodent brain [49]. Recently, we have found that in the frontal cortex of prenatally stressed animals, an increase in the IRS-1 phosphorylation of Ser312 occurs, which results in an inhibition of IRS-1 activity [4]. Serine phosphorylation is known to be involved in the inhibitory regulation of IRS-1 tyrosine phosphorylation [50], which would explain the decrease in the level of tyrosine-phosphorylated IRS-1 observed in the frontal cortex of prenatally stressed animals. Some reports have also described the role of serine phosphorylation in IRS-2 inactivation [51], which results in an impairment of hippocampal LTP [52].

Taken together, these data indicate that behavioral changes evoked by prenatal stress coexist with glutamatergic transmission and synaptic plasticity alterations in the motor cortex of adult rats. These effects would compromise the plastic capacity of the motor system. The exact mechanism responsible for the observed effects requires further studies.

## References

[1] Charil A, Laplante DP, Vaillancourt C, King S. Prenatal stress and brain development. Brain Res Rev. 2010;65: 56–79. 10.1016/j.brainresrev.2010.06.002 20550950

[2] Maccari S, Krugers HJ, Morley-Fletcher S, Szyf M, Brunton PJ. The consequences of early-life adversity: neurobiological, behavioural and epigenetic adaptations. J Neuroendocrinol. 2014;26: 707–723. 10.1111/jne.12175 25039443

[3] Markham JA, Koenig JI. Prenatal stress: role in psychotic and depressive diseases. Psychopharmacology (Berl). 2011;214: 89–106. 10.1007/s00213-010-2035-0 20949351 PMC3050113

[4] Basta-Kaim A, Szczesny E, Glombik K, Slusarczyk J, Trojan E, Tomaszewski KA, et al Prenatal stress leads to changes in IGF-1 binding proteins network in the hippocampus and frontal cortex of adult male rat. Neuroscience. 2014;274: 59–68. 10.1016/j.neuroscience.2014.05.010 24857711

[5] Basta-Kaim A, Szczesny E, Glombik K, Stachowicz K, Slusarczyk J, Nalepa I, et al Prenatal stress affects insulin-like growth factor-1 (IGF-1) level and IGF-1 receptor phosphorylation in the brain of adult rats. Eur Neuropsychopharmacol. 2014;24: 1546–1556. 10.1016/j.euroneuro.2014.07.002 25106693

[6] Budziszewska B, Szymanska M, Leskiewicz M, Basta-Kaim A, Jaworska-Feil L, Kubera M, et al The decrease in JNK- and p38-MAP kinase activity is accompanied by the enhancement of PP2A phosphate level in the brain of prenatally stressed rats. J Physiol Pharmacol. 2010;61: 207–215. 20436222

[7] Mairesse J, Silletti V, Laloux C, Zuena AR, Giovine A, Consolazione M, et al Chronic agomelatine treatment corrects the abnormalities in the circadian rhythm of motor activity and sleep/wake cycle induced by prenatal restraint stress in adult rats. Int J Neuropsychopharmacol. 2013;16: 323–338. 10.1017/S1461145711001970 22310059

[8] Szymanska M, Budziszewska B, Jaworska-Feil L, Basta-Kaim A, Kubera M, Leśkiewicz M, et al The effect of antidepressant drugs on the HPA axis activity, glucocorticoid receptor level and FKBP51 concentration in prenatally stressed rats. Psychoneuroendocrinology. 2009;34: 822–832. 10.1016/j.psyneuen.2008.12.012 19195790

[9] Son GH, Geum D, Chung S, Kim EJ, Jo JH, Kim CM, et al Maternal stress produces learning deficits associated with impairment of NMDA receptor-mediated synaptic plasticity. J Neurosci. 2006;26: 3309–3318. 16554481 10.1523/JNEUROSCI.3850-05.2006 PMC6674110

[10] Yang J, Han H, Cao J, Li L, Xu L. Prenatal stress modifies hippocampal synaptic plasticity and spatial learning in young rat offspring. Hippocampus. 2006;16: 431–436. 16598704 10.1002/hipo.20181

[11] Yaka R, Salomon S, Matzner H, Weinstock M. Effect of varied gestational stress on acquisition of spatial memory, hippocampal LTP and synaptic proteins in juvenile male rats. Behav Brain Res. 2007;179: 126–132. 17320196 10.1016/j.bbr.2007.01.018

[12] Yeh CM, Huang CC, Hsu KS. Prenatal stress alters hippocampal synaptic plasticity in young rat offspring through preventing the proteolytic conversion of pro-brain-derived neurotrophic factor (BDNF) to mature BDNF. J Physiol. 2012;590: 991–1010. 10.1113/jphysiol.2011.222042 22155932 PMC3381323

[13] Lemaire V, Koehl M, Le Moal M, Abrous DN. Prenatal stress produces learning deficits associated with an inhibition of neurogenesis in the hippocampus. Proc Natl Acad Sci USA. 2000;97: 11032–11037. 11005874 10.1073/pnas.97.20.11032 PMC27143

[14] Fujioka A, Fujioka T, Ishida Y, Maekawa T, Nakamura S. Differential effects of prenatal stress on the morphological maturation of hippocampal neurons. Neuroscience. 2006;141: 907–915. 16797130 10.1016/j.neuroscience.2006.04.046

[15] Mychasiuk R, Gibb R, Kolb B. Prenatal stress alters dendritic morphology and synaptic connectivity in the prefrontal cortex and hippocampus of developing offspring. Synapse. 2012;66: 308–314. 10.1002/syn.21512 22121047

[16] Kolb B, Mychasiuk R, Muhammad A, Li Y, Frost DO, Gibb R. Experience and the developing prefrontal cortex. Proc Natl Acad Sci USA. 2012;109 Suppl 2:17186–17193. 10.1073/pnas.1121251109 23045653 PMC3477383

[17] Donoghue JP, Wise SP. The motor cortex of the rat: cytoarchitecture and microstimulation mapping. J Comp Neurol. 1982;212: 76–88. 6294151 10.1002/cne.902120106

[18] Sanes JN, Donoghue JP. Plasticity and primary motor cortex. Annu Rev Neurosci. 2000;23: 393–415. 10845069 10.1146/annurev.neuro.23.1.393

[19] Kleim JA, Barbay S, Nudo RJ. Functional reorganization of the rat motor cortex following motor skill learning. J Neurophysiol. 1998;80: 3321–3325. 9862925 10.1152/jn.1998.80.6.3321

[20] Remple MS, Bruneau RM, VandenBerg PM, Goertzen C, Kleim JA. Sensitivity of cortical movement representations to motor experience: evidence that skill learning but not strength training induces cortical reorganization. Behav Brain Res. 2001;123: 133–141. 11399326 10.1016/s0166-4328(01)00199-1

[21] Rioult-Pedotti MS, Friedman D, Hess G, Donoghue JP. Strengthening of horizontal cortical connections following skill learning. Nat Neurosci. 1998;1: 230–234. 10195148 10.1038/678

[22] Rioult-Pedotti MS, Friedman D, Donoghue JP. Learning-induced LTP in neocortex. Science (Wash). 2000;290: 533–536. 11039938 10.1126/science.290.5491.533

[23] Monfils MH, Teskey GC. Skilled-learning-induced potentiation in rat sensorimotor cortex: a transient form of behavioural long-term potentiation. Neuroscience. 2004;125: 329–336. 15062976 10.1016/j.neuroscience.2004.01.048

[24] Detke MJ, Rickels M, Lucki I. Active behaviors in the rat forced swimming test differentially produced by serotonergic and noradrenergic antidepressants. Psychopharmacology (Berl). 1995;121: 66–72. 8539342 10.1007/BF02245592

[25] Wabno J, Hess G. Repeated administration of imipramine modifies GABAergic transmission in rat frontal cortex. J Neur Trans. 2013;120: 711–719. 10.1007/s00702-012-0919-3 23180303 PMC3631518

[26] Bobula B, Hess G. Antidepressant treatments-induced modifications of glutamatergic transmission in rat frontal cortex. Pharmacol Rep. 2008;60: 865–871. 19211978

[27] Bobula B, Wabno J, Hess G. Imipramine counteracts corticosterone-induced enhancement of glutamatergic transmission and impairment of long-term potentiation in rat frontal cortex. Pharmacol Rep. 2011;63: 1404–1412. 22358088 10.1016/s1734-1140(11)70704-6

[28] Hamani C, Diwan M, Macedo CE, Brandão ML, Shumake J, Gonzalez-Lima F, et al Antidepressant-like effects of medial prefrontal cortex deep brain stimulation in rats. Biol Psychiatry. 2010;67: 117–124. 10.1016/j.biopsych.2009.08.025 19819426

[29] Duncan GE, Johnson KB, Breese GR. Topographic patterns of brain activity in response to swim stress: assessment by 2-deoxyglucose uptake and expression of Fos-like immunoreactivity. J Neurosci. 1993;13: 3932–3943. 8366353 10.1523/JNEUROSCI.13-09-03932.1993 PMC6576454

[30] Stoltz S, Humm JL, Schallert T. Cortical injury impairs contralateral forelimb immobility during swimming: a simple test for loss of inhibitory motor control. Behav Brain Res. 1999;106: 127–132. 10595428 10.1016/s0166-4328(99)00100-x

[31] Bobula B, Tokarski K, Hess G. Repeated administration of antidepressants decreases field potentials in rat frontal cortex. Neuroscience. 2003;120: 765–769. 12895516 10.1016/s0306-4522(03)00380-4

[32] Hess G, Jacobs KM, Donoghue JP. N-methyl-D-aspartate receptor mediated component of field potentials evoked in horizontal pathways of rat motor cortex. Neuroscience. 1994;61: 225–235. 7969904 10.1016/0306-4522(94)90226-7

[33] Hess G, Aizenman CD, Donoghue JP. Conditions for the induction of long-term potentiation in layer II/III horizontal connections of the rat motor cortex. J Neurophysiol. 1996;75: 1765–1778. 8734579 10.1152/jn.1996.75.5.1765

[34] Tokarski K, Bobula B, Wabno J, Hess G. Repeated administration of imipramine attenuates glutamatergic transmission in rat frontal cortex. Neuroscience. 2008;153: 789–795. 10.1016/j.neuroscience.2008.03.007 18403127

[35] Simkus CR, Stricker C. Properties of mEPSCs recorded in layer II neurones of rat barrel cortex. J Physiol. 2002;545: 509–520. 12456830 10.1113/jphysiol.2002.022095 PMC2290708

[36] Li H, Prince DA. Synaptic activity in chronically injured, epileptogenic sensory-motor neocortex. J Neurophysiol. 2002;88: 2–12. 12091528 10.1152/jn.00507.2001

[37] Sun H, Guan L, Zhu Z, Li H. Reduced levels of NR1 and NR2A with depression-like behavior in different brain regions in prenatally stressed juvenile offspring. PLoS One. 2013;8: e81775 10.1371/journal.pone.0081775 24278457 PMC3835745

[38] Zhang XH, Jia N, Zhao XY, Tang GK, Guan LX, Wang D, et al Involvement of pGluR1, EAAT2 and EAAT3 in offspring depression induced by prenatal stress. Neuroscience. 2013;250: 333–341. 10.1016/j.neuroscience.2013.04.031 23694703

[39] Schulz PE. Long-term potentiation involves increases in the probability of neurotransmitter release. Proc Natl Acad Sci USA. 1997;94: 5888–5893. 9159170 10.1073/pnas.94.11.5888 PMC20876

[40] Sáez I, Friedlander MJ. Plasticity between neuronal pairs in layer 4 of visual cortex varies with synapse state. J Neurosci. 2009;29: 15286–15298. 10.1523/JNEUROSCI.2980-09.2009 19955381 PMC2824571

[41] Tokarski K, Bobula B, Kusek M, Hess G. The 5-HT_7_ receptor antagonist SB 269970 counteracts restraint stress-induced attenuation of long-term potentiation in rat frontal cortex. J Physiol Pharmacol. 2011;62: 663–667. 22314569

[42] Maccari S, Darnaudery M, Van Reeth O. Hormonal and behavioural abnormalities induced by stress in utero: an animal model for depression. Stress. 2001;4: 169–181. 22432138 10.3109/10253890109035016

[43] Morley-Fletcher S, Darnaudery M, Koehl M, Casolini P, Van Reeth O, Maccari S. Prenatal stress in rats predicts immobility behavior in the forced swim test. Effects of a chronic treatment with tianeptine. Brain Res. 2003;989: 246–251. 14556947 10.1016/s0006-8993(03)03293-1

[44] Szczesny E, Slusarczyk J, Glombik K, Budziszewska B, Kubera M, Lason W, et al Possible contribution of IGF-1 to depressive disorder. Pharmacol Rep. 2013;65, 1622–1631. 24553010 10.1016/s1734-1140(13)71523-8

[45] Russo VC, Gluckman PD, Feldman EL, Werther GA. The insulin-like growth factor system and its pleiotropic function in the brain. Endocr Rev. 2005;6: 916–943. 10.1210/er.2004-0024 16131630

[46] Molina DP, Ariwodola OJ, Weiner JL, Brunso-Bechtold JK, Adams MM. Growth hormone and insulin-like growth factor-I alter hippocampal excitatory synaptic transmission in young and old rats. Age (Dordr). 2013;35: 1575–1587. 10.1007/s11357-012-9460-4 22851280 PMC3776110

[47] Fernandez AM, Torres-Alemán I. The many faces of insulin-like peptide signalling in the brain. Nat Rev Neurosci. 2012;13: 225–239. 10.1038/nrn3209 22430016

[48] Sun XJ, Rothenberg P, Kahn CR, Backer JM, Araki E, Wilden PA, et al Structure of the insulin receptor substrate IRS-1 defines a unique signal transduction protein. Nature. 1991;352: 73–77. 1648180 10.1038/352073a0

[49] White MF. IRS proteins and the common path to diabetes. Am J Physiol Endocrinol Metab. 2002;283: E413–422. 12169433 10.1152/ajpendo.00514.2001

[50] Tanti JF, Jager J. Cellular mechanisms of insulin resistance: role of stress-regulated serine kinases and insulin receptor substrates (IRS) serine phosphorylation. Curr Opin Pharmacol. 2009;9: 753–762. 10.1016/j.coph.2009.07.004 19683471

[51] Moloney AM, Griffin RJ, Timmons S, O'Connor R, Ravid R, O'Neill C. Defects in IGF-1 receptor, insulin receptor and IRS-1/2 in Alzheimer's disease indicate possible resistance to IGF-1 and insulin signalling. Neurobiol Aging. 2010;31: 224–243. 10.1016/j.neurobiolaging.2008.04.002 18479783

[52] Martín ED, Sánchez-Perez A, Trejo JL, Martin-Aldana JA, Cano Jaimez M, Pons S, et al IRS-2 Deficiency impairs NMDA receptor-dependent long-term potentiation. Cereb Cortex. 2012;22: 1717–1727. 10.1093/cercor/bhr216 21955917 PMC3388895

